# A comprehensive scoring system for the diagnosis and staging of adhesive capsulitis: development, application, and implications

**DOI:** 10.1007/s00590-024-04098-z

**Published:** 2024-09-28

**Authors:** Fabio Vita, Danilo Donati, Roberto Tedeschi, Marco Miceli, Paolo Spinnato, Flavio Origlio, Enrico Guerra, Marco Cavallo, Salvatore Massimo Stella, Luigi Tarallo, Giuseppe Porcellini, Stefano Galletti, Cesare Faldini

**Affiliations:** 1 IRCCS Istituto Ortopedico Rizzoli, 1st Orthopaedics and Traumatology clinic, Bologna, University of Bologna, Bologna, Italy; 2 Department of Biomedical and Neuromotor Sciences (DIBINEM), Alma Mater Studiorum, University of Bologna, Via Zamboni 33, 40126 Bologna, Italy; 3 Diagnostic and Interventional Radiology, IRCCS Istituto Ortopedico Rizzoli, Bologna, Italy; 4 Physical Therapy and Rehabilitation Unit, IRCCS Rizzoli Orthopedic Institute, University of Bologna, Bologna, Italy; 5 Shoulder & Elbow Surgery Department, IRCCS Istituto Ortopedico Rizzoli, Bologna, Italy; 6 Musculoskeletal Ultrasound School, Italian Society for Ultrasound in Medicine and Biology, Bologna, Italy; 7 Department of Clinical and Experimental Medicine, SIUMB Advanced School for Musculoskeletal Ultrasound, University Post-Graduate Course, Santa Chiara University Hospital, Pisa, Italy; 8 Orthopedic and Traumatology Department of Sassuolo, University of Modena and Reggio Emilia, Modena, Italy; 9 Physical Therapy and Rehabilitation Unit, Policlinico Di Modena, Modena, Italy; 10 Clinical and Experimental Medicine PhD Program, University of Modena and Reggio Emilia, Modena, Italy; 11 Department of Orthopaedics and Traumatology, University of Modena and Reggio Emilia, Policlinico di Modena, Modena, Italy

**Keywords:** Adhesive capsulitis, Frozen shoulder, Shoulder pain, Ultrasound imaging, Clinical evaluation

## Abstract

**Introduction:**

Adhesive capsulitis (AC), often referred to as frozen shoulder, presents a diagnostic challenge due to its insidious onset and progressive nature. The condition is characterized by pain and restricted motion in the shoulder, with a predilection for individuals between 40 and 60 years of age. A novel scoring system was developed to enhance the accuracy of diagnosing AC and distinguishing between its stages, aiming to streamline clinical decision-making and treatment planning.

**Methods:**

A cohort of patients with symptoms suggestive of AC was assessed using the new scoring system, which integrates clinical, radiological, and patient history factors. Parameters included comorbidities like diabetes mellitus, recent immobility, rotator cuff tears, and specific ultrasound findings. Patients were scored and categorized into definitive AC, uncertain diagnosis, or exclusion from AC, with scores > 7, 6–2, and < 2, respectively.

**Results:**

The scoring system effectively categorized patients, with those scoring > 7 demonstrating pronounced symptoms and ultrasound changes consistent with Phase 2 AC. Patients with scores between 6 and 2 were classified into uncertain Phase 1 or Phase 3, necessitating further observation. Scores < 2 effectively excluded AC, indicating a need to explore alternative diagnoses.

**Conclusion:**

The structured scoring system demonstrated potential as a comprehensive tool for diagnosing AC. By quantitatively assessing a range of contributory factors, it allowed for the stratification of the disease into distinct stages. This system is anticipated to improve early diagnosis and the precision of treatment interventions, although further validation in larger cohorts is warranted.

**Level of evidence:**

II-III.

## Introduction

Adhesive capsulitis (AC), also known as “frozen shoulder,” is a debilitating condition characterized by pain and restricted mobility in the shoulder joint. Initially described by Codman in 1934 and later coined “adhesive capsulitis” by Neviaser in 1945, the condition is marked by a progressive loss of shoulder movement due to fibrosis and contracture of the glenohumeral joint capsule [20, 22]. Despite its clear clinical presentation, the precise etiology and natural progression of AC remain poorly understood. Predominantly affecting individuals between 40 and 60 years of age, AC can severely impact daily activities and quality of life [27].

AC is classified into primary (idiopathic) and secondary forms, with the latter associated with identifiable risk factors, such as neurological issues, rotator cuff injuries, diabetes, arthritis, recent fractures, immobility of the upper extremity, heart conditions, or autoimmune diseases [8, 28]. The condition evolves through three distinct phases—freezing, frozen, and thawing—each characterized by varying degrees of pain and mobility limitation [21].

Diagnosis of AC relies on a comprehensive clinical examination complemented by imaging techniques like ultrasound (US) and magnetic resonance imaging (MRI) to exclude other potential causes of shoulder pain. Ultrasound, in particular, offers an accessible and cost-effective diagnostic alternative, with specific findings such as capsular thickening serving as key indicators of the condition [1, 25, 26].

Treatment modalities for AC are diverse, ranging from physical therapy to more invasive procedures like manipulation under anesthesia, ultrasound-guided capsular distension, and surgical interventions. Among these, hydrodistension has shown promise in providing swift pain relief and improved joint function [11]. Physical therapy remains integral in restoring mobility and strength [9, 31].

The absence of specific diagnostic tests and biomarkers complicates early detection and diagnosis of AC, underscoring the need for reliable diagnostic criteria. This study aims to develop a simple, yet effective scoring system for AC that not only aids in diagnosis but also helps distinguish between its early and advanced stages [10, 18, 19]. By integrating ultrasound findings, pathological history, and clinical evaluation, this scoring system seeks to facilitate early identification and initiation of appropriate treatment strategies, potentially mitigating the condition’s progression to more severe stages.

## Ethical issues

This cross-sectional, interventional observational study received approval from the local Ethics Committee (379/2022/Sper/IOR), underscoring our commitment to ethical research practices. Conducted in accordance with the ethical standards delineated in the Declaration of Helsinki, this study sought to ensure the utmost respect for participant rights and well-being throughout its duration. Prior to any data collection or intervention, informed consent was diligently obtained from all participants. This process included a comprehensive explanation of the study’s objectives, the nature of the interventions involved, and how the data would be utilized for research purposes. Participants were assured of their anonymity and the confidentiality of their personal information, aligning with ethical guidelines for research involving human subjects.

## Materials and methods

We integrated the study on the scoring system with the research on management via hydrodistension to evaluate not only the diagnostic efficacy of the system but also its clinical utility in guiding therapeutic decisions.

Between September 1st, 2022, and April 28th, 2023, a prospective evaluation was conducted on 40 patients diagnosed with adhesive capsulitis (Table 1). These patients underwent comprehensive clinical and radiographic assessments including anteroposterior and axillary shoulder x-rays conducted by a senior orthopedic surgeon specializing in shoulder pathology. The purpose of these evaluations was to exclude other conditions presenting with similar symptoms, such as calcific tendinopathy and glenohumeral osteoarthritis, ensuring an accurate diagnosis of adhesive capsulitis. Patients were excluded from the study if they were pregnant, reported allergies to anesthetics, had a diagnosis other than adhesive capsulitis, or were unable to provide informed consent.

**Table 1:** Demographic and Clinical Characteristics of Patients with Adhesive Capsulitis

Variable mean ± SD or n(%)	AC patients (n = 40)
Age (years)	53.3 ± 10.7
Body mass index	26.1 ± 4.2
Male:Female	12:28
*Education*	
Primary school	0 (0)
Secondary school	3 (10)
High school	17 (40)
University degree	20 (50)
*Work activities*	
Employee	24 (65)
Professional	9 (20)
Retiree	7 (15)

Within this study, we developed and introduced a simplified scoring system aimed at enhancing the diagnostic process for adhesive capsulitis. This innovative system, outlined in Table 1, incorporates a range of factors, including comorbidities, results from clinical assessments, and ultrasound parameters. The scoring system is designed to classify the diagnosis of adhesive capsulitis into distinct phases based on cumulative scores: Phase 2 is identified for scores greater than 7, Phase 1 or Phase 3 for scores ranging between 6 and 2, and a score lower than 2 leads to the exclusion of the pathology. This structured approach allows for a nuanced understanding of the condition’s severity and assists in formulating customized treatment strategies for each patient.

This table summarizes the demographic and clinical characteristics of the 40 patients diagnosed with adhesive capsulitis (AC) included in the study. Variables include age, body mass index (BMI), gender distribution, education level, and work activities. Data are presented as mean ± standard deviation (SD) for continuous variables and as numbers and percentages for categorical variables.

The score involves the evaluation of:

## Comorbidities

The consideration of comorbidities plays a pivotal role in the comprehensive assessment of Adhesive Capsulitis (AC). The patient’s medical history can significantly influence the diagnostic process, as certain conditions may either support or negate the likelihood of AC. This nuanced approach to diagnosis is reflected in the scoring system designed to aid in the confirmation or exclusion of adhesive capsulitis.

### Supporting comorbidities

Certain conditions, when present in a patient’s history, are indicative of an increased likelihood of developing adhesive capsulitis. These include:

- Diabetes Mellitus: Patients with diabetes are at a higher risk for developing AC due to the systemic nature of the disease, which can affect collagen in the shoulder joint capsule.
- Recent Shoulder Fractures: Injuries to the shoulder, such as fractures, can initiate a cascade of inflammatory responses, leading to capsular fibrosis.

Each of these conditions contributes 0.5 points to the scoring system. Their presence in a patient’s medical history thus supports the likelihood of an AC diagnosis.

### Negating comorbidities

Conversely, certain conditions can significantly decrease the probability of an AC diagnosis, each deducting 5 points from the score:

- Autoimmune Diseases: Conditions that systemic inflammation may mask or contribute to shoulder stiffness, but are not directly indicative of AC.
- Calcification of Tendinopathies: Calcific tendinitis can cause pain and limited movement, symptoms similar to AC but with a different underlying pathology.
- Glenohumeral Arthritis: Osteoarthritis of the shoulder can mimic the symptoms of AC but involves degenerative changes to the joint surfaces rather than capsular contracture.
- Fracture of Upper Limb: Recent immobility of the upper limb was considered as a single factor with a score of 0.5, as it significantly contributes to the development of adhesive capsulitis.
- Rotator Cuff Tear: This condition can cause pain and limited range of motion, leading to confusion with AC but stemming from a different pathology.
- Neurological Diseases: Neurological conditions affecting the shoulder can mimic the stiffness and pain of AC but are caused by neural rather than mechanical constraints.
- Subacromial Bursitis (SAD): While it can cause shoulder pain and reduced mobility, it’s a distinct condition from AC and involves inflammation of the bursa, not capsular fibrosis.

The presence of any of these negating factors leads to a significant reduction in the score, effectively excluding the diagnosis of adhesive capsulitis based on their impact.

## Clinical evaluation

In the diagnosis of Adhesive Capsulitis (AC), clinical evaluation plays a crucial role [17], particularly in assessing the duration and evolution of pain, as well as the patient’s perception of improvement or deterioration over time. This nuanced approach helps differentiate AC from other conditions with similar symptoms and contributes to the scoring system designed to aid in diagnosis. Acute pain is defined as pain persisting for less than one month, while chronic pain is considered when it has been present for more than one month. The latter is more characteristic of the progression of adhesive capsulitis and is assigned a higher score (1 point) compared to acute pain (0.5 points) [2, 6, 16]. The patient’s subjective perception of improvement or worsening of symptoms over the past two weeks was quantified based on the perceived intensity of stiffness and pain. A perceived improvement is indicative of a resolving phase and is assigned a score of 0, while worsening, which aligns with the disease’s progression, is given a score of 1.

### Pain duration

- Acute Phase (< 1 month): If the patient reports pain duration of less than 1 month, this is considered the acute phase. Such short-term pain can be indicative of a variety of shoulder pathologies, including shoulder calcifications or periarthritis, which may present similarly to the early stages of AC. Consequently, a score of 0.5 is assigned in these cases, reflecting the ambiguous nature of the symptom and its potential attribution to conditions other than AC.
- Chronic Pain (> 1 month): Pain persisting for more than 1 month suggests a transition into chronicity. Chronic pain is more characteristic of AC, especially as it aligns with the condition’s progression into stages of increased stiffness and decreased range of motion. However, it’s also important to consider that other pathologies previously excluded, such as glenohumeral arthrosis, may also present with chronic pain. Therefore, chronic pain merits a closer look and supports a higher suspicion of AC.

### Clinical improvement perception

- Sensation of Improvement: The patient’s subjective sensation of improvement or worsening over the previous two weeks provides valuable insight into the pathology’s evolution. If the patient perceives an improvement, this could indicate the condition is not progressing toward the characteristic worsening associated with the freezing or frozen stages of AC, thus receiving a score of 0. This suggests a potentially different pathology or a less severe stage of AC [29, 30].
- Sensation of Worsening: Conversely, a perceived worsening of symptoms is characteristic of AC’s natural progression, where the condition histologically evolves, typically reflecting an increase in joint stiffness and pain. This perception aligns with the disease’s pathology and is therefore given 1 point in the scoring system, signaling a higher probability of AC.

## Shoulder mobility assessment

The evaluation of shoulder mobility plays a pivotal role in diagnosing adhesive capsulitis, a condition characterized by progressive stiffness and pain in the shoulder joint. To systematically assess shoulder mobility and its implications for adhesive capsulitis, we have adopted a scoring system aligned with the guidelines provided by the American Academy of Orthopedic Surgeons (AAOS). This system quantifies the degree of limitation in specific shoulder movements, offering valuable insights into the severity of the condition.

### Elevation (vertical arm raise)

- 0 to 30 degrees: Assigned a score of 3, reflecting significant restriction in movement.
- 30 to 60 degrees: Score of 2, indicating moderate limitation.
- 60 to 90 degrees: Score of 1, denoting limited but slightly improved mobility.
- 90 to 120 degrees: Score of 0.5, representing minimal movement restriction.
- 120 to 160 degrees: Score of 0, suggesting negligible or no limitation.
- Above 160 degrees: Also scored as 0, indicating full mobility.

### Abduction (lateral arm movement)

- 0 to 30 degrees: Score of 3, signifying severe movement restriction.
- 30 to 60 degrees: Score of 2, indicating moderate limitation.
- 60 to 90 degrees: Score of 1, reflecting limited but somewhat improved movement.
- 90 to 120 degrees: Score of 0.5, representing minimal limitation.
- 120 to 160 degrees: Score of 0, suggesting little or no restriction.
- Above 160 degrees: Also scored as 0, indicative of unrestricted movement.

### Abduction internal rotation

- 0 to 30 degrees: Assigned a score of 2, indicating significant limitation in internal rotation.
- 30 to 60 degrees: Score of 1, suggesting moderate restriction in internal rotation capability.
- 60 to 90 degrees: Score of 0.5, reflecting limited but improved internal rotation.

### Abduction external rotation

- 0 to 30 degrees: Score of 2, signifying substantial restriction in external rotation.
- 30 to 60 degrees: Score of 1, indicating moderate limitation in external rotation.
- 60 to 90 degrees: Score of 0.5, denoting a limited but improved capacity for external rotation.

## Ultrasound evaluation

Ultrasound evaluation is a cornerstone in the diagnosis of Adhesive Capsulitis (AC), providing invaluable insights that complement clinical assessments. High-resolution ultrasound imaging facilitates the visualization of a range of shoulder pathologies, offering a dynamic, cost-effective, and non-invasive diagnostic tool. For patients with suspected or confirmed AC, ultrasound studies focus on specific anatomical features that serve as critical indicators of the condition. These features include the coracohumeral ligament (CHL) thickness, rotator interval (RI) characteristics, anterior/axillary region (AR) changes, and effusion in the long head of the biceps tendon (LHBT) sheath.

1. Coracohumeral Ligament (CHL) Thickness:
  Patients are evaluated in a seated position, with the shoulder neutral and the hand resting on the thigh. The ultrasound transducer is placed obliquely on the lateral edge of the coracoid process to image the CHL longitudinally [14]. Rotational movements help identify the CHL, which folds or stretches with internal or external rotation, respectively. A thickened CHL, especially when compared to the contralateral limb, is indicative of AC and contributes a score of 0.5.
2. Rotator Interval (RI) Thickening and Hypervascularity:
  The RI is inspected in an oblique axial plane, with the patient’s fist held laterally. Both B-mode and power Doppler ultrasound assess RI thickness, defined as the distance between the long head of the biceps tendon and the surrounding fat, including the CHL. Thickening and hypervascularity of the RI, relative to the contralateral side, add a score of 1.
3. Thickening in the Anterior/Axillary Regions (AR):
  Dynamic ultrasound assessment of the anterior shoulder capsule reveals limitations in motion and thickening that could restrict the subscapularis muscle–tendon unit’s range of motion. Such stiffness or thickening, particularly when it alters the external rotation phase or the visualization of the posterior capsule-synovial recess, is compared with the contralateral shoulder. An increase in AR thickness > 0.5 mm is significant and scored as 1.5, while an absence of thickening scores 0 [5, 7, 23].
4. Effusion of the Long Head of the Biceps Tendon (LHBT) Sheath:
  The synovial sheath around the LHBT often communicates with the glenohumeral joint, making it a key area for detecting joint effusions. Dynamic evaluation focuses on the interaction between the tendon and its stabilizing structures. The presence of LHBT sheath effusion, indicative of potential capsular fibrosis or synovitis, is scored as 1. Its absence scores 0 [13, 24].

This ultrasound-based scoring system enhances the diagnostic accuracy for AC, allowing for a detailed examination of the shoulder’s anatomical and functional status. By quantifying changes in key structures, clinicians can better identify AC, differentiate it from other conditions, and tailor treatment strategies accordingly [4].

### Ultrasound-guided infiltrative treatment

Ultrasound-guided infiltrative treatment, particularly hydrodistension, is a minimally invasive therapeutic approach for managing adhesive capsulitis. This technique utilizes ultrasound imaging to accurately guide the injection of therapeutic agents into the glenohumeral joint, ensuring precise delivery to the affected area [10]. The procedure is described as follows:

1. Patient Positioning: The patient is positioned prone, which facilitates easier access to the shoulder joint for the injection process.
2. Ultrasound Equipment: A high-frequency linear transducer, ranging from 5 to 17 MHz, is employed to provide clear, real-time images of the shoulder’s internal structures. This visualization ensures that the needle is accurately guided to the intended site within the glenohumeral joint.
3. Needle Specification: A 90 mm long, 20-gauge (G) needle is used for the procedure. The size and length of the needle are chosen to optimize the accuracy of the injection while minimizing discomfort for the patient.
4. Injection Technique: The injection is performed using a latero to medial posterior approach to access the shoulder joint. This pathway is chosen based on its safety and the ease with which it allows the needle to reach the glenohumeral joint under ultrasound guidance.
5. Injection Composition:
  o Cortisone: 1 ml of cortisone (depomedrol 40 mg/ml) is injected to reduce inflammation within the joint. Cortisone is a powerful anti-inflammatory agent that can significantly alleviate pain and swelling.
  o Lidocaine Hydrochloride: 10 ml of 2% lidocaine hydrochloride is used as a local anesthetic to provide immediate pain relief in the area of the injection.
  o Saline: 10 ml of saline is injected to distend the joint capsule. The hydrodistension process involves the injection of fluid to stretch the joint capsule, which can help to break up adhesions and improve range of motion.

The combination of these agents serves multiple purposes: cortisone reduces inflammation, lidocaine provides pain relief, and saline facilitates joint capsule distension, collectively addressing the key aspects of adhesive capsulitis pathology. This procedure is performed under ultrasound guidance to ensure the safety and efficacy of the treatment, minimizing risks associated with blind injections.

## Results

The scoring system presented in Table 1 provides a structured diagnostic framework for Adhesive Capsulitis (AC), employing a holistic approach by integrating data from anamnestic records, clinical evaluation, pain assessment, range of motion (ROM) analysis, and ultrasound findings (Table 2). This comprehensive scoring mechanism allows for the classification of patients into distinct diagnostic categories, each reflecting the severity and stage of AC. Below is an overview of the categorization based on cumulative scores:

1. Definitive Diagnosis of Adhesive Capsulitis (“Yes”—Stage 2):
  o A total score greater than 7 unequivocally indicates a diagnosis of AC. Patients classified under this stage exhibit pronounced clinical symptoms, significant functional limitations, intense pain, restricted ROM, and ultrasound findings consistent with AC.
  o In our study, patients diagnosed with Stage 2 AC underwent glenohumeral hydrodistension treatment, with some receiving additional bursal infiltration. All were subjected to a physiotherapy rehabilitation protocol tailored to address the specific challenges and needs posed by the condition.
2. Uncertain Diagnosis (Phase 1 or Phase 3):
  o Scores ranging from 6 to 2 indicate an uncertain diagnosis, positioning the patient in a transitional phase of the condition. This ambiguity in diagnosis suggests the potential onset (Phase 1) or resolution phase (Phase 3) of AC, necessitating further diagnostic assessments or continuous monitoring to reach a conclusive determination.
  o Treatment for patients within this uncertain diagnostic category typically involves hydrodistension, with bursal infiltration being a less common adjunct. A physiotherapy rehabilitation protocol is also employed to aid recovery and mitigate symptoms.
3. Exclusion of AC (“No”):
  o A score below 2 effectively rules out the diagnosis of AC, indicating that the patient does not exhibit the significant clinical, functional, or imaging features characteristic of AC.
  o This finding underscores the absence of AC and directs clinicians toward alternative diagnoses or treatment pathways that better align with the patient’s symptoms and presentation.

**Table 2:** Diagnostic scoring system for adhesive capsulitis (Frozen shoulder)

Comorbidities	Yes	No
Diabete Mellitus	0.5	0
Recent Immobility/Fracture Upper Limb	0.5	0
Rotator Cuff Tear	− 5	0
Tendinophaty Calcify	− 5	0
Arthrosis Glenohumeral	− 5	0
Neurological Disease of the Shoulder	− 5	0
Effusion SAD	− 5	0

Clinical Evaluation	Yes	No
Acute Pain	0.5	0
Chronic Pain > 1 month	1	0
Feel Better or Worse in the last 2 weeks	0	1

Range of Movement	0°–30°	30°–60°	60°–90°	90°–120°	120°–160°	> 160°
Elevation	3	2	1	0.5	0	0
Abduction	3	2	1	0.5	0	0
Intrarotation in abduction	2	1	0.5	0	0	0
Extarotation in abduction	2	1	0.5	0	0	0

Ultrasound Evaluation	Yes	No
RI thickening and hypervascularity	1	0
Thickening AR	1.5	0
Thickening CHL Ligament	0.5	0
Effusion of LHBT sheath	1	0

Table 1 outlines a comprehensive scoring system to assist in the diagnosis and staging of adhesive capsulitis, commonly known as frozen shoulder. The system integrates various factors including comorbidities, clinical evaluation criteria, range of motion measurements, and ultrasound findings. Each parameter is assigned a score that collectively determines the diagnosis: a total score greater than 7 indicates a definitive phase 2 diagnosis (Yes), scores between 6 and 2 suggest an uncertain status (phase 1 or phase 3), and scores less than 2 are indicative of the absence of the condition (No). Negative scores are attributed to conditions that typically rule out adhesive capsulitis, while positive scores reflect characteristics commonly associated with the condition. This scoring approach aims to provide a nuanced assessment, promoting targeted treatment strategies based on the identified phase of adhesive capsulitis

The application of this scoring system to patients at their initial visit proved instrumental in guiding treatment decisions and providing insights into the potential progression of the pathology. In our comprehensive evaluation of patients with suspected Adhesive Capsulitis (AC), a variety of clinical and sonographic parameters were meticulously recorded and analyzed. Our study included patients with a range of motion assessments for flexion (ranging from 50 to 170 degrees, mean 107.6 ± 29.4 degrees), abduction (ranging from 20 to 170 degrees, mean 80.1 ± 37.2 degrees), internal rotation (ranging from 0 to 75 degrees, mean 35.7 ± 18.3 degrees), and external rotation (ranging from 0 to 75 degrees, mean 29.8 ± 19.7 degrees).

The patient cohort demonstrated a notable prevalence of diabetes mellitus (DM) at 25% (10 out of 40 patients), aligning with established literature that associates DM with an increased risk of developing AC. Recent immobility, another factor considered in the assessment of AC, was reported in 20% of the cases.

An analysis of pain revealed that 40% (16 out of 40) of patients experienced acute pain, while a larger proportion, 60% (24 out of 40), reported chronic pain. The subjective assessment of the condition’s trajectory showed that 45% (18 out of 40) of patients felt their symptoms had worsened, referred to as ‘Worse’ in the data, whereas 15% (6 out of 40) reported improvement, categorized as ‘Better’.

Ultrasonographically, the effusion in the long head biceps tendon (LHBT) sheath was observed in 55% (22 out of 40) of the patients, suggesting its potential role as an indicator of AC. Thickening of the anterior recess (AR) was recorded in 62.5% (25 out of 40), thickening of the coracohumeral ligament (CHL) in 75% (30 out of 40), and thickening in the rotator interval (RI) in 32.5% (13 out of 40) of the patients.

The application of our scoring system led to the categorization of patients into definitive diagnosis (Stage 2 of AC) with a mean score of 10.5 for those scoring above 7, suggesting severe impairment. The uncertain diagnosis category (scores between 6 and 2) had a mean score of 5.2, indicating a milder presentation or early resolution phase. Those with scores below 2, suggesting exclusion from an AC diagnosis, had a mean score of 1.8 and were subsequently considered for alternative diagnostic pathways.

Overall, the results highlight the multifactorial nature of AC and the utility of an integrated scoring system to assess and stratify patients, potentially informing targeted therapeutic interventions.”

## Discussion

The advancement of an Adhesive Capsulitis (AC) scoring system represents a significant innovation in the clinical approach to a condition that has historically presented diagnostic challenges. Our scoring system amalgamates comorbidities, clinical assessments, and ultrasound findings to provide a multidimensional diagnostic framework, thus enabling the stratification of AC into its early and advanced stages.

Early detection of AC is paramount for initiating effective treatment protocols. The literature corroborates that timely interventions can alter disease progression and improve functional outcomes [20]. Our scoring system is structured to reflect this paradigm, facilitating early diagnosis by incorporating a spectrum of clinical presentations and objective findings into a cohesive model.

AC, characterized by insidious onset and progressive loss of shoulder motion, presents a spectrum of clinical manifestations. This diversity necessitates a diagnostic tool capable of capturing the variable presentation of the disease. The scoring system we propose allows for such differentiation, offering a granular assessment that stratifies patients according to the accumulated severity reflected in their cumulative scores. This stratification is crucial, as it informs the choice of therapeutic intervention, which must be tailored to the individual’s specific disease stage [25].

The scoring system’s integration of comorbid conditions is aligned with evidence suggesting that systemic diseases such as diabetes mellitus are implicated in the pathogenesis of AC, possibly through glycosylation-induced collagen cross-linking and subsequent capsular stiffness [1]. Similarly, immobility and shoulder trauma have been recognized as risk factors for the development of AC due to their association with inflammatory cascades and capsular fibrosis [20]. Conversely, the negative scoring for conditions like autoimmune diseases and glenohumeral arthrosis serves to refine the specificity of the AC diagnosis, in line with differential diagnostic considerations emphasized in current rheumatological guidelines [8].

The mobility assessment within the scoring system correlates with the functional limitation extent and is thus instrumental in guiding the clinical diagnosis of AC. It quantifies the physical examination findings, facilitating a more objective interpretation of the ROM deficits encountered. This quantification aligns with the American Academy of Orthopaedic Surgeons’ recommendations for standardized shoulder assessment protocols.

Incorporation of sonographic parameters adds a layer of diagnostic precision.

The relative weights assigned to each criterion in the scoring system were determined based on a review of existing literature and expert consensus among clinical specialists. The subjectivity in score formulation was mitigated through an in-depth evaluation of available evidence and discussions among orthopedic specialists [3, 12, 32].

Ultrasound’s ability to non-invasively visualize soft tissue structures in real-time provides an indispensable adjunct to the clinical assessment [10]. Parameters such as coracohumeral ligament thickness and joint capsule changes have been implicated in the pathophysiology of AC, reflecting inflammatory and fibrotic processes [14].

In light of these considerations, the scoring system developed in our study not only streamlines the diagnosis of AC but also anchors it in a pathophysiological context, enhancing the clinician’s ability to prognosticate and manage the disease effectively.

### Limitations of the study

It is crucial, however, to acknowledge the study’s limitations. Primarily, the modest cohort size, while sufficient for preliminary inquiry, may introduce an element of statistical bias, thereby limiting the generalizability of the findings. The sampling frame may not fully represent the broader population afflicted by AC, and as such, our scoring system’s performance in wider clinical practice remains to be rigorously tested. The score of 0.5 assigned to diabetes mellitus was determined considering the recognized but variable role of this comorbidity in the pathogenesis of adhesive capsulitis. Studies have shown a significant, though not absolute, correlation between diabetes and the severity of adhesive capsulitis, justifying a moderate weight in the overall score [15].

## Conclusions

Our scoring system emerges as a significant contribution to the field, offering a potential shift in the diagnostic approach to adhesive capsulitis. It underscores the necessity of a multifaceted diagnostic strategy that can adapt to the complexity of this condition. The need for further validation through larger-scale studies is clear; only through such research can we ensure that the proposed scoring system holds its promise in clinical practice, leading to improved patient outcomes in the management of AC.
